# Steering the methanol steam reforming reactivity of intermetallic Cu–In compounds by redox activation: stability *vs.* formation of an intermetallic compound–oxide interface†

**DOI:** 10.1039/d1cy00913c

**Published:** 2021-07-23

**Authors:** Kevin Ploner, Andrew Doran, Martin Kunz, Albert Gili, Aleksander Gurlo, Nicolas Köwitsch, Marc Armbrüster, Johannes Bernardi, Maximilian Watschinger, Simon Penner

**Affiliations:** Department of Physical Chemistry, University of Innsbruck Innrain 52c A-6020 Innsbruck Austria simon.penner@uibk.ac.at +4351250758003; Advanced Light Source, Lawrence Berkeley National Laboratory Berkeley California 94720 USA; Chair of Advanced Ceramic Materials, Institut für Werkstoffwissenschaften und – Technologien, Technical University Berlin Hardenbergstr. 40 D-10623 Berlin Germany; Institute of Chemistry, Technical University Berlin Sekretariat TC 8, Straße des 17. Juni 124 D-10623 Berlin Germany; Institute of Chemistry, Materials for Innovative Energy Concepts, Technical University Chemnitz Straße der Nationen 62 D-09111 Chemnitz Germany; University Service Centre for Transmission Electron Microscopy, TU Wien Wiedner Hauptstr. 8-10 A-1040 Vienna Austria

## Abstract

To compare the inherent methanol steam reforming properties of intermetallic compounds and a corresponding intermetallic compound–oxide interface, we selected the Cu–In system as a model to correlate the stability limits, self-activation and redox activation properties with the catalytic performance. Three distinct intermetallic Cu–In compounds – Cu_7_In_3_, Cu_2_In and Cu_11_In_9_ – were studied both in an untreated and redox-activated state resulting from alternating oxidation–reduction cycles. The stability of all studied intermetallic compounds during methanol steam reforming (MSR) operation is essentially independent of the initial stoichiometry and all accordingly resist substantial structural changes. The inherent activity under batch MSR conditions is highest for Cu_2_In, corroborating the results of a Cu_2_In/In_2_O_3_ sample accessed through reactive metal–support interaction. Under flow MSR operation, Cu_7_In_3_ displays considerable deactivation, while Cu_2_In and Cu_11_In_9_ feature stable performance at simultaneously high CO_2_ selectivity. The missing significant self-activation is most evident in the *operando* thermogravimetric experiments, where no oxidation is detected for any of the intermetallic compounds. *In situ* X-ray diffraction allowed us to monitor the partial decomposition and redox activation of the Cu–In intermetallic compounds into Cu0.9In0.1/In_2_O_3_ (from Cu_7_In_3_), Cu_7_In_3_/In_2_O_3_ (from Cu_2_In) and Cu_7_In_3_/Cu0.9In0.1/In_2_O_3_ (from Cu_11_In_9_) interfaces with superior MSR performance compared to the untreated samples. Although the catalytic profiles appear surprisingly similar, the latter interface with the highest indium content exhibits the least deactivation, which we explain by formation of stabilizing In_2_O_3_ patches under MSR conditions.

## Abstract 2

To compare the properties of intermetallic compounds and intermetallic compound–oxide interfaces, Cu–In was used as a model to correlate stability limits, self-activation and redox activation with the inherent methanol steam reforming performance.

## Graphical abstract

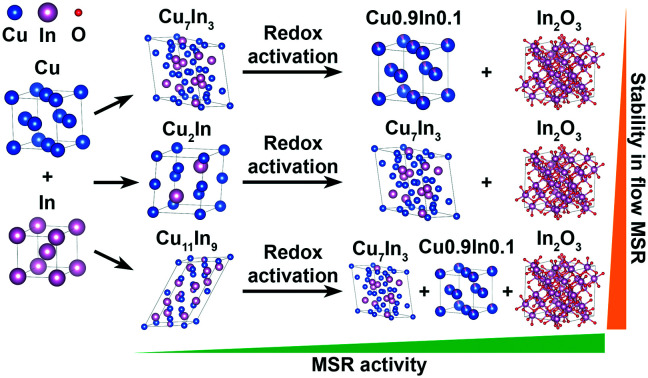

## 1. Introduction

Methanol represents a promising candidate for the reversible storage and release of hydrogen, which is itself a suitable carrier of renewable energy. It is liquid at ambient conditions and, thus, easier to implement into the existing fuel infrastructure.^1,2^ For the efficient and fast on-demand release of hydrogen, which can be utilized in a proton exchange membrane fuel cell (PEMFC) in automotive applications, methanol steam reforming is the most desirable reforming reaction with the highest H_2_ yield.^1,3,4^ For the application of on-board MSR and the direct use of the reformate in a PEMFC, several criteria have to be satisfied.^1^ The catalysts have to be optimized with respect to activity, long-term stability and selectivity toward MSR (eqn (1)):^3,5^

1 CH_3_OH_(g)_ + H_2_O_(g)_ ⇄ 3H_2,(g)_ + CO_2,(g)_Δ*H*_r_^0^ = 49.6 kJ mol^−1^

Competing side reactions lead to the formation of electrode-poisoning CO, where only a concentration in the low ppm regime is considered to be tolerable in most fuel cells.^6,7^ Therefore, CO-forming reactions like methanol dehydrogenation (eqn (2)) or the reverse water–gas shift reaction (eqn (3)) have to be avoided.^3,5^

2 CH_3_OH_(g)_ ⇄ 2H_2,(g)_ + CO_(g)_Δ*H*_r_^0^ = 90.6 kJ mol^−1^

3 CO_2,(g)_ + H_2,(g)_ ⇄ H_2_O_(g)_ + CO_(g)_Δ*H*_r_^0^ = 41.1 kJ mol^−1^

A Cu/ZnO/Al_2_O_3_ catalyst that is mainly utilized for the reverse reaction, *i.e.*, methanol synthesis, is currently the only commercially available system for MSR applications.^8^ It provides a comparably high activity, but suffers from deactivation by progressive metallic Cu particle sintering and produces a too high level of CO for direct use of the reformate in a PEMFC without an additional cleaning step.^3,8^ Hence, alternative materials must be explored to provide a catalyst fulfilling all requirements.

Intermetallic compounds (IMCs) have recently gained increasing attention in MSR, especially systems based on Pd.^9–14^ High emphasis has been given to the increasing dynamics of the intermetallic compound structure during catalytic operation, eventually leading to their partial or full decomposition and the formation of an IMC–oxide or metal–oxide interface.^15,16^ The formation of the intermetallic compound (or metal)–oxide interface can be triggered *via* two main routes.^15^ (i) Deliberate preparation of the interface by reactive metal–support interaction, *i.e.*, a catalytic pre-treatment usually involving reduction treatments of a conventional metal–oxide system in hydrogen. Many specific IMC–oxide systems, *e.g.* ZnPt/ZnO,^17^ ZnPd/ZnO,^16^ PdGa/Ga_2_O_3_,^18,19^ PdIn/In_2_O_3_,^20^ or PtIn/In_2_O_3_,^17^ can be accessed through this preparation routine.^21^ (ii) *In situ* self-activation of an IMC precursor in the reaction mixture.^22–25^ In some cases, the decomposition can also be triggered by targeted pre-treatments.^15^ As an illustrative example, showing the potential of this approach, we recently reported self-activation of a bimetallic Cu_51_Zr_14_/Cu precursor during MSR operation.^22,23^ The resulting active state, composed of metallic Cu nanoparticles embedded in an oxidized tetragonal ZrO_2_ matrix, displayed high activity and CO_2_ selectivity in MSR.^22,23^

The specific Cu–In system has already been in the focus of research due to the combination of Cu as a methanol activator with an element whose oxide (resulting from potential decomposition) also exhibits a promising methanol steam reforming performance. In_2_O_3_ was studied regarding its catalytic properties in methanol synthesis by CO_2_ hydrogenation, as well as MSR, exhibiting a promising performance in both reactions.^4,26,27^ Cu is well known to be active in MSR and is a constituent in many high-performance MSR catalysts.^3,8,22^ Hydrogen reduction of a CuO/In_2_O_3_ precursor at different temperatures allowed us to access two distinct states, *i.e.*, metallic Cu on In_2_O_3_ and Cu_2_In on In_2_O_3_. Hence, it was possible to assess the MSR performance of the specific Cu_2_In/In_2_O_3_ interface, which exhibited an enhanced activity at a similar CO_2_ selectivity, compared to Cu/In_2_O_3_.^28^

The present study aims at elucidating the specific stability limits of several selected intermetallic Cu–In compounds of different stoichiometry during methanol steam reforming operation and assessing the respective self-activation capability *vs.* redox activation by selected oxidation and reduction treatments. This will allow assessing the intrinsic activity of the respective IMCs and directly relating it to the MSR performance of the potentially resulting metallic Cu (or intermetallic Cu–In compound)–oxide interface.

To accomplish this task, three exemplary intermetallic Cu–In compounds, Cu_7_In_3_, Cu_2_In and Cu_11_In_9_, were synthesized as bulk materials by high-temperature synthesis and their MSR performance in the as-synthesized state was assessed. Subsequently, we conducted targeted oxidation and reduction pre-treatments prior to catalytic operation to assess the reactivity differences. Monitoring of the structural changes with respect to bulk stability of the IMCs, surface chemistry and segregation behavior was based on *in situ* X-ray diffraction (XRD), *operando* thermogravimetric analysis, quasi *in situ* X-ray photoelectron spectroscopy and electron microscopy combined with activity/selectivity and long-term stability measurements in methanol steam reforming.

## 2. Experimental

### 2.1. Synthesis of the intermetallic compounds

We prepared the IMCs by melting metallic Cu (ChemPUR, 99.999%) and In (ChemPUR, 99.999%). The educts were weighed and mixed in an Ar-filled glovebox according to the target stoichiometry and subsequently transferred to quartz-glass ampules. Evacuation to a final pressure below 10^−5^ mbar and sealing using a hydrogen/oxygen flame followed. On the basis of the Cu–In phase diagram,^29,30^ the Cu_7_In_3_ sample was annealed at 600 °C for 30 days and Cu_2_In, as well as Cu_11_In_9_, at 270 °C for 90 days. The resulting reguli were crushed to obtain samples for catalytic testing and the associated analyses.

### 2.2. Catalytic testing

#### 2.2.1. Recirculating batch reactor

This setup for characterization of the catalyst performance is specialized for small sample amounts (≈10–100 mg) and the detection of trace by-products due to its small reactor volume (≈13.8 ml). Continuous quantification of the gas phase composition is ensured by a quadrupole mass spectrometer (QMS, Balzers QMG 311) connected to the reactor *via* a capillary leak. The entire sample compartment consists of quartz glass and can be heated with a Linn High Term furnace up to 1100 °C. The temperature is monitored with a K-type thermocouple (NiCr–Ni) placed close to the catalyst.

The supply of the MSR mixture is achieved by a liquid solution of methanol and water prepared in a ratio to obtain an equilibrium gas phase composition of methanol : water = 1 : 2 at room temperature. Gas and liquid phase were purified by three consecutive freeze–pump–thaw cycles before the mixture was expanded into the pre-evacuated reactor. Each MSR reaction (termed “MSR400” with 400 °C denoting the final isothermal temperature) was conducted by introducing the mixture (≈28 mbar) into the reactor at 100 °C (to avoid condensation), followed by the addition of Ar (≈6 mbar). Ar is used for intensity correction of all mass signals with respect to thermal expansion and the steady gas withdrawal through the capillary to the mass spectrometer. To enhance the recirculation efficiency, as well as the heat transfer between the gas phase and the catalyst, the reactor is finally backfilled with He to 1 bar total pressure.

After baseline and Ar intensity correction, external calibration of the relevant gases, including the relative fragmentation patterns (*e.g.*, for the *m*/*z* = 28 fragment of CO_2_), enables the calculation of reactant partial pressures. Differentiation of the partial pressures with respect to time yields formation rates in mbar min^−1^. Application of the ideal gas law and normalization to the copper mass gives the specific activities in μmol g_Cu_^−1^ s^−1^ to ensure direct literature comparability.^3^ Normalization to the accessible number of Cu surface sites, which would yield turnover frequencies (assuming that Cu is the active site) could not be performed, since established methods for the determination of the specific Cu surface area, like dissociative N_2_O adsorption, are not feasible for intermetallic Cu–In compounds.

The integral CO_2_ selectivity was calculated by dividing the partial pressure of CO_2_ by the sum of the partial pressures of CO_2_, CO and CH_4_. Values larger than 1, caused by slight variation of the baseline at small values of the sum (leading to huge spikes in the integral CO_2_ selectivity) were set to 1. The methanol conversion is expressed relative to the *m*/*z* = 31 intensity at the start of the temperature program.

The apparent activation energy (*E*_a_) of CO_2_ formation was determined by plotting the specific activity in μmol g_Cu_^−1^ s^−1^*vs.* the temperature in K. The corresponding profile is fitted with the Arrhenius function below conversions of 10%. As the simultaneous independent fits of *A* and *E*_a_ yielded values ranging from 1.1 × 10^7^ μmol g_Cu_^−1^ s^−1^ to 2.1 × 10^8^ μmol g_Cu_^−1^ s^−1^ for the pre-exponential factor, *A* was fixed at 10^8^ μmol g_Cu_^−1^ s^−1^ for enhanced relative comparability of the obtained values for the apparent activation energy.

#### 2.2.2. Continuous flow reactor

A fixed-bed reactor (PID Eng&Tech, Microactivity Reference) was employed for continuous flow experiments. It is connected to a MicroGC (Varian CP 4900, equipped with a 10 m back-flushed M5A column, a 20 m back-flushed M5A column and a 10 m PPU column, Agilent Technologies) for simultaneous quantification of H_2_, CO, CO_2_ and CH_4_. The catalysts were diluted with graphite (ChemPUR, <100 μm, 99.9%) to ensure a homogeneous gas flow through the sample and placed on top of a quartz-glass fleece in the reactor tube (stainless steel coated with silicon oxide, inner diameter 7.9 mm). For the MSR experiments, a 1 : 1 molar H_2_O/MeOH mixture (0.01 ml min^−1^ DI-H_2_O_(l)_, 0.0225 ml min^−1^ MeOH_(l)_, Fisher Scientific, HPLC grade) was loaded onto a 10% He in N_2_ carrier gas (45 ml min^−1^, Air Liquide, 99.999%) by quantitative evaporation. The unreacted vapors were removed from the gas phase by condensation in a cooling trap and the gas stream was dried with a Nafion® membrane with a counter flow of 100 ml min^−1^ N_2_. The gas phase composition was analyzed by online gas chromatography, yielding the specific activity toward H_2_, CO, CO_2_ and CH_4_. The CO_2_ selectivity was obtained by division of the specific activity of CO_2_ by the sum of the specific activities of CO, CO_2_ and CH_4_.

### 2.3. X-ray diffraction (XRD)

#### 2.3.1. *Ex situ* powder XRD

A Stadi P diffractometer (STOE & Cie GmbH, Darmstadt, Germany) in transmission geometry was employed for *ex situ* powder XRD experiments. It is equipped with a MYTHEN2 DCS6 detector system (DECTRIS Ltd., Switzerland) and a Mo X-ray tube (GE Sensing & Inspection Technologies GmbH, Ahrensburg, Germany) operated at 50 kV and 40 mA. A curved Ge(111) monochromator crystal selects the Mo K_α1_ radiation with a wavelength of 0.7093 Å. Data evaluation was performed with the WinX^POW^ software^31^ and phase analysis was based on reference diffractograms either retrieved from the ICDD database^32^ or calculated using the software *VESTA* 3 (ref. 33) and the corresponding cif-files.

#### 2.3.2. *In situ* powder XRD

The evolution of the sample bulk structure under oxidizing and reducing conditions was studied with synchrotron-based *in situ* powder XRD at beamline 12.2.2 of the Advanced Light Source (ALS) at the Lawrence Berkeley National Laboratory (LBNL). The employed setup has been described in detail elsewhere.^34,35^ The diffraction patterns were recorded in transmission mode with a DECTRIS PILATUS3 detector (collecting one pattern per 30 s) and utilizing a monochromatic beam with a spot size of ≈20 μm and an energy of 25 keV. The Dioptas software^36^ was used for radial integration of the 2D detector images, yielding diffraction patterns, as well as calibration of the exact wavelength (0.4905 Å), the sample-to-detector distance (345 mm) and the detector tilt by measuring and analyzing the LaB_6_ NIST standard reference material (SRM) 660b.

The crushed sample (≈1 mg) was placed at the bottom of a quartz-glass capillary (700 μm in diameter) that was inserted into a SiC sleeve, which was heated with two infrared lights. The required gases were supplied by Alicat mass flow controllers and directed to the heated samples through another capillary (for details see ref. 34). The oxidative treatments were conducted with a gas mixture of 2 ml min^−1^ O_2_ and 8 ml min^−1^ He and applying a heating ramp from 25–800 °C with 10 °C min^−1^, an isothermal period of 30 min and cooling to 25 °C with 20 °C min^−1^. Reduction was accomplished in 4 ml min^−1^ pure H_2_ and a temperature program with heating from 25–500 °C with 10 °C min^−1^, an isothermal period of 30 min and cooling to 25 °C with 20 °C min^−1^ was executed.

### 2.4. *Operando* thermogravimetric analysis (*operando* TGA)

TGA/MS characterizations under MSR conditions were performed with a Netzsch STA 449F3 Jupiter in Al_2_O_3_ crucibles using a defined amount of ground sample. Each measurement was corrected by a reference experiment with an identical temperature program in the same gas atmosphere with empty Al_2_O_3_ crucibles. A mass spectrometer (Pfeiffer, Omnistar GSD 301 O3) was employed for monitoring of selected mass/charge signals characteristic for MSR. At the start of the *operando* TG experiments, the samples were heated in 40 ml min^−1^ He flow to 150 °C with a rate of 5 °C min^−1^ and kept at that temperature for 15 min. Then, 0.39 g h^−1^ of liquid methanol/water mixture (50 mol% methanol (Fisher Scientific, HPLC grade) and 50 mol% deionized water), which was continuously evaporated at 200 °C, was loaded onto the He stream. Successively, the temperature was increased to 600 °C in this atmosphere with a rate of 5 °C min^−1^, followed by an isothermal period of 60 min and cooling to 300 °C with 5 °C min^−1^. At 300 °C the flow of the MSR mixture was stopped and cooling to room temperature was continued.

## 3. Results and discussion

### 3.1. Sample synthesis and characterization of Cu_7_In_3_, Cu_2_In and Cu_11_In_9_

**Fig. 1:**
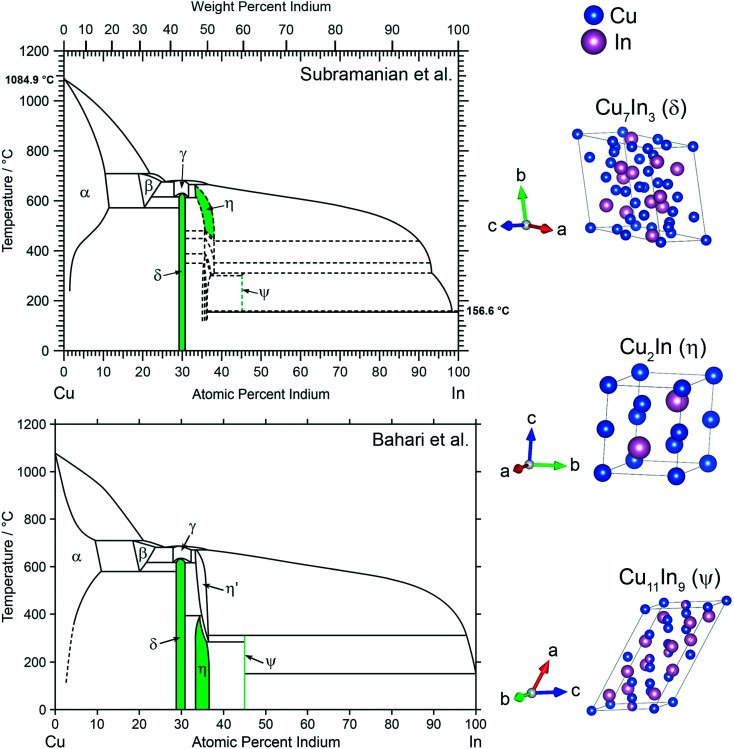
Phase diagram of Cu and In adapted from Subramanian *et al.*^29^ at the top and the version adapted from Bahari *et al.*^30^ at the bottom including the synthesized compounds of this study highlighted in green. The crystal structures of Cu_7_In_3_, Cu_2_In and Cu_11_In_9_ are illustrated on the right.^39–41^

Based on a previous study regarding the reactive metal–support interaction of Cu and In_2_O_3_, where the Cu_2_In–oxide interface particularly stood out in MSR performance,^28^ three intermetallic compounds, Cu_2_In and two adjacent to this stoichiometry, were selected in this work. Two versions of the Cu–In phase diagram, one provided by Subramanian *et al.*^29^ and the other by Bahari *et al.*,^30^ are depicted in Fig. 1. The are two main differences between the two versions of the Cu–In phase diagram. The first concerns the room temperature stability of Cu_2_In and Cu_11_In_9_, which is proposed by Bahari *et al.*^30^ and supported by data from Bolcavage *et al.*^37^ The second difference is the existence of several phases in the region of Cu_2_In according to Subramanian *et al.*^29^ and Jain *et al.*,^38^ who proposed five phases in this region, as opposed to the two phases given by Bahari *et al.*^30^ and Bolcavage *et al.*^37^ The IMCs synthesized in this study are marked in green in both versions of the phase diagram and are listed in Table 1. Cu_7_In_3_ (denoted as δ phase in Fig. 1) exhibits a triclinic crystal structure (space group *P*1̄) and a homogeneity range of 2 at%.^39^ The latter is also true for Cu_2_In (η phase in Fig. 1), a high-temperature phase according to Subramanian *et al.*,^29^ but stable at room temperature according to Bahari *et al.*,^30^ with a hexagonal structure (space group *P*6_3_/*mmc*).^40^ Several other compounds at this composition have been proposed by Subramanian *et al.*^29^ and Jain *et al.*^38^ around this composition, indicated by the dashed lines in Fig. 1 in the phase diagram at the top. A more In-rich compound is monoclinic Cu_11_In_9_ (ψ phase in Fig. 1, space group *C*2/*m*),^41^ which exhibits no homogeneity range according to both versions of the phase diagram.

**Table 1:** List of prepared intermetallic Cu–In compounds

Phase	Nominal stoichiometry	Sample acronym	Copper content/at%	Copper content/wt%
δ	Cu_7_In_3_	CuIn70	70.00	56.36
η	Cu_2_In	CuIn67	66.67	52.54
ψ	Cu_11_In_9_	CuIn55	55.00	40.35

The unambiguous identification of the phase composition of intermetallic Cu–In compounds and their decomposition products is a generally highly complex task due to two reasons. Firstly, the most intense reflections of the three investigated phases as well as other intermetallic compounds – *e.g.* Cu_1.535_In,^42^ Cu_1.568_In,^42^ Cu_1.81_In,^43^ Cu_10_In_7_,^44^ Cu_4_In (ref. 40) and Cu_9_In_4_ (ref. 40) – are located between 18.5° and 19.5° 2*θ* (*λ* = 0.7093 Å), including the alloy Cu0.9In0.1.^45^ The latter exhibits the same space group as metallic Cu and the incorporation of indium leads to lattice expansion, shifting the most intense reflection to 2*θ* ≈ 19.1° (*λ* = 0.7093 Å). This renders the assignment of potential trace phases difficult. Secondly, the Cu–In phase diagram provided by Subramanian *et al.*,^29^ supported by a study of Jain *et al.*,^38^ suggests the existence of several yet uncharacterized phases at a similar composition as Cu_2_In, introducing additional complexity and uncertainty to any phase assignment. For these reasons, acronyms for the respective stoichiometries of the samples were defined (see Table 1).

### 3.2. MSR performance and structural evolution of the untreated intermetallic compounds

**Fig. 2:**
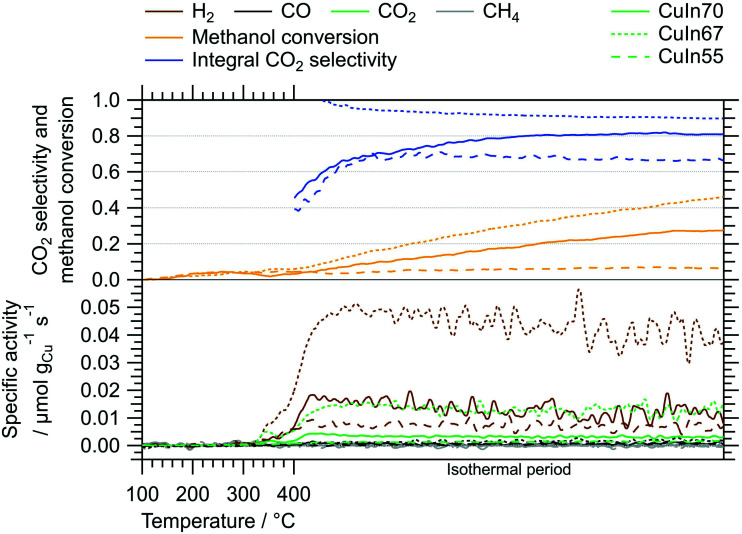
MSR profiles of CuIn70, CuIn67, CuIn55 measured between 100 °C and 400 °C including an isothermal period of 170 min at 400 °C. Color code: orange – methanol conversion, blue – integral CO_2_ selectivity, specific activity of brown: H_2_, black: CO, green: CO_2_, gray: CH_4_. Heating rate: 5 °C min^−1^; sample mass: CuIn70 – 47.2 mg, CuIn67 – 51.8 mg, CuIn55 – 51.5 mg.

Fig. 2 depicts the MSR performance of the untreated crushed catalysts. As a general result, the low specific surface area of the crushed IMCs translates into a correspondingly low specific mass activity of all samples, especially compared to conventional powder catalysts. Based on the sieve fraction of 20–32 μm employed in the MSR flow measurements, the specific surface area can be estimated to lie between 0.020 m^2^ g^−1^ and 0.035 m^2^ g^−1^, assuming spherical particles. While the onset temperatures of H_2_ and CO_2_ formation (both ≈320 °C) are almost independent of the Cu–In stoichiometry, the intermediate composition, CuIn67, exhibits the best MSR performance in terms of specific activity, integral CO_2_ selectivity and methanol conversion. Due to the generally low activity, the determination of the CO_2_ selectivity is very sensitive to baseline fluctuations and the specific activity toward CO is close to the detection limit (especially for CuIn55).

**Fig. 3:**
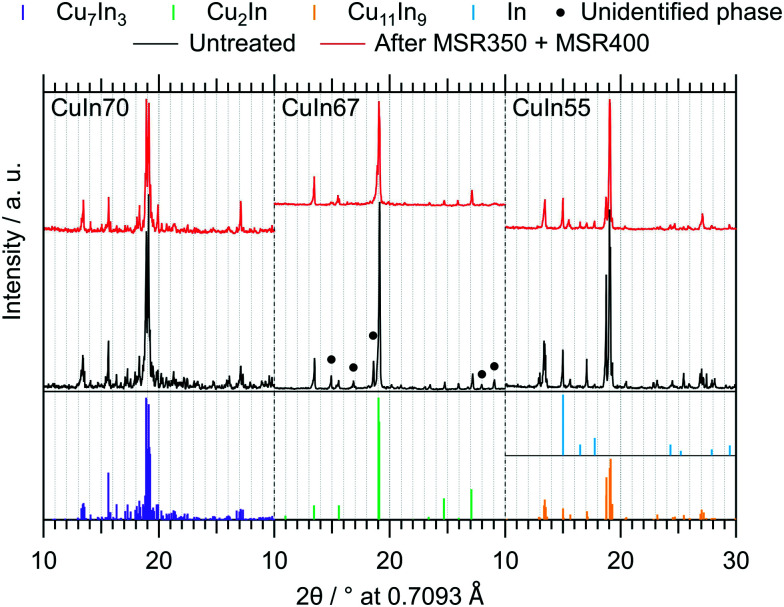
*Ex situ* collected XRD patterns of the untreated IMCs and after two MSR cycles, the first up to 350 °C and the second up to 400 °C. The references for Cu_7_In_3_,^39^ Cu_2_In,^40^ Cu_11_In_9_ (ref. 41) and In (ref. 46) were calculated with the software *VESTA* 3 (ref. 33) using the respective cif-files (*λ* = 0.7093 Å).

The *ex situ* collected XRD patterns of the untreated intermetallic compounds, as well as their structural state after MSR, are shown in Fig. 3. CuIn70 is present as single-phase Cu_7_In_3_ after synthesis. After MSR, Cu_7_In_3_ is remarkably stable, as we detect no significant decomposition products such as crystalline In_2_O_3_ or Cu (oxide). CuIn67 is mainly represented by Cu_2_In.^40^ In line with the Cu–In phase diagram of Subramanian *et al.*,^29,38^ unassignable phases were present before contact to the MSR reaction mixture, but vanished during MSR. Cu_2_In remained almost unaltered. CuIn55 was obtained as Cu_11_In_9_ according to the XRD pattern, containing traces of metallic In as by-product. Upon MSR, intensification of the reflections assigned to metallic indium occurred, accompanied by an associated decrease in the Cu_11_In_9_ reflections. Since we detect no In_2_O_3_ or Cu (oxide) and less intense Cu_11_In_9_ reflections are visible after MSR, a partial transformation to a Cu-richer IMC with higher symmetry (caused by the segregation of indium) could account for these changes in the diffraction pattern.

**Fig. 4:**
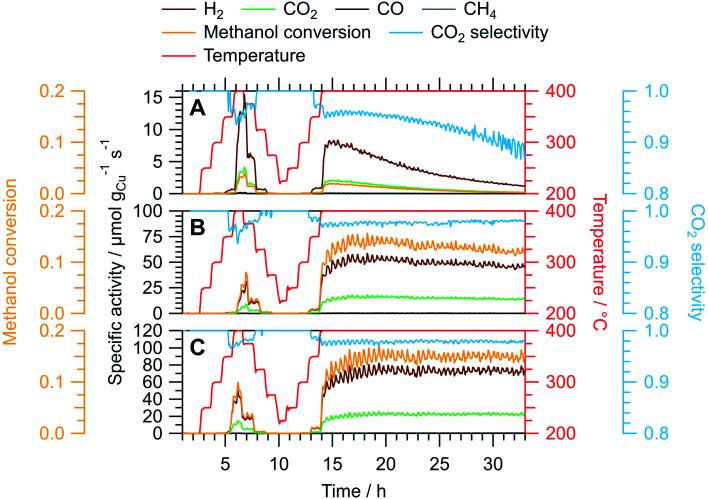
Continuous flow MSR measurements using the untreated samples CuIn70 (panel A), CuIn67 (panel B) and CuIn55 (panel C). The dynamic fraction (0–14 h on-stream) consists of temperature steps from 200 °C to 400 °C, followed by an intermediate cooling before increasing the temperature to 400 °C again for an isothermal long-term performance test. Color code: orange – methanol conversion, blue – CO_2_ selectivity, red – temperature, specific activity of brown: H_2_, black: CO, green: CO_2_, gray: CH_4_. GHSV: 1800 h^−1^; sample mass: CuIn70 – 150.0 mg, CuIn67 – 150.0 mg, CuIn55 – 150.1 mg.

We assessed the activation and long-term stability of CuIn70, CuIn67 and CuIn55 under MSR operation using catalytic continuous flow measurements (Fig. 4). CuIn70 exhibits the smallest specific activity toward H_2_ formation, the worst CO_2_ selectivity and displays rapid deactivation on-stream (from initially 14 μmol g_Cu_^−1^ s^−1^ at 400 °C in the dynamic temperature steps to approximately 1 μmol g_Cu_^−1^ s^−1^ at the end of the isothermal period lasting 19 h) accompanied by a decrease in the CO_2_ selectivity. On the contrary, CuIn67 exhibits an activation behavior observed as an increase in the specific activity of H_2_ formation from the dynamic 400 °C step (≈30 μmol g_Cu_^−1^ s^−1^) to the start of the isothermal period (≈54 μmol g_Cu_^−1^ s^−1^) at a stable CO_2_ selectivity of approximately 98%. From 17 h to 32 h on-stream, a slight decrease in the specific activity of H_2_ formation from ≈54 μmol g_Cu_^−1^ s^−1^ to ≈45 μmol g_Cu_^−1^ s^−1^ and the methanol conversion from 14% to 12% is visible. CuIn55 shows a similar behavior as CuIn67, where the specific activity of H_2_ formation increases from ≈42 μmol g_Cu_^−1^ s^−1^ at 400 °C in the dynamic segment to ≈74 μmol g_Cu_^−1^ s^−1^ after a total of 19 h on-stream in the isothermal period. No significant deactivation can be observed for CuIn55, which displays both a stable methanol conversion of 15% and CO_2_ selectivity of 98%. As a rule, the catalysts were cooled before the steady-state operation to minimize changes of the material during catalytic steady-state operation. In that way, pre-heating the catalyst to higher temperatures changes the material faster (especially the “healing of edges”, which is referred to as sintering in the manuscript below) and then a lower steady-state operation temperature does not lead to such changes anymore.

The utilization of both batch and flow measurements grants access to complementary information on the catalytic behavior of the samples. In each reactor, the amount of MSR mixture with which the catalysts are in contact with is considerably different, resulting in a distinct activation/deactivation behavior. Therefore, we interpret the MSR performance observed in the recirculating batch reactor as an initial state that is already transformed in the continuous flow reactor before its catalytic implications arise through significant formation rates. Hence, the deactivation of CuIn70 is not visible in the batch reactor, while the activation of CuIn55 is not yet complete. An attentive reader will notice apparent differences in the hydrogen production especially for CuIn55 between the recirculating batch and the flow measurements. We attribute this essentially to the very rich redox chemistry occurring on intermetallic compounds in MSR, which is not only temperature-dependent, but also depends upon conversion (*i.e.*, the change of the redox potential of the MSR atmosphere with conversion). In addition, oxidic species are very likely to be involved in the catalytic cycle. Small differences, as different total pressure or the removal/non-removal of products will have an influence on the mechanism of the reaction at the atomic level, manifesting itself differently in the catalytic characterization.

**Fig. 5:**
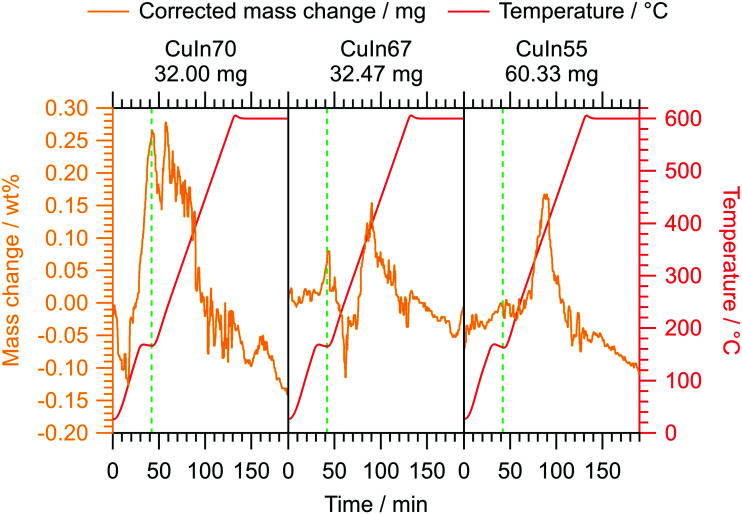
*Operando* TGA characterization of CuIn70, CuIn67 and CuIn55 under continuous flow MSR conditions. The temperature program involves heating from 160 °C to 600 °C at a rate of 5 °C min^−1^ and includes an isothermal period of 60 min. As a guide to the eye, we marked the introduction of the MSR mixture by vertical dashed green lines for each measurement.

In addition to the characterization of the MSR performance in a continuous flow setup, we conducted *operando* TGA under similar conditions on the untreated samples. The relative mass change of all samples does not exceed 0.3 wt%, highlighting the general high stability of the intermetallic Cu–In compounds under MSR conditions. At low temperature, we observe an intermediary reversible mass increase, probably related to a transient oxidation step. It proceeds at lower temperatures for CuIn70 compared to CuIn67 and CuIn55. At the final temperature of 400 °C, the mass change of CuIn70 has decreased once again to 0.02 wt% (maximum mass increase 0.28 wt% at 230 °C), while the maximum values of CuIn67 (0.15 wt% at 400 °C) and CuIn55 (0.17 wt% at 380–395 °C) are approached approximately at 400 °C. The *operando* TGA experiments help to interpret the catalytic performance tests: CuIn70 displays a mass decrease at temperatures below 400 °C, which we ascribe to a reduction of the previously formed oxide phases responsible for the initial mass increase. Assuming, that only indium oxidation occurs, this would yield 1.6 wt% In_2_O_3_ for CuIn70, 0.9 wt% In_2_O_3_ for CuIn67 and 1.0 wt% In_2_O_3_ for CuIn55 at maximum. This suggests that CuIn70 is fully reduced during the isothermal period in the flow MSR measurements and therefore more prone to deactivation by sintering (see Fig. 4). In this regard, sintering does not only refer to the reduction of the total specific surface area, but also to the reduction of the number of potentially reactive edge sites by rounding or “healing” of the particles. This process starts at the Hüttig temperature, which is the border for surface mobility and can be estimated as 0.3 times the melting temperature in Kelvin.^47^*Vice versa*, as the maxima of mass increase for CuIn67 and CuIn55 are located around 400 °C, a prolonged exposure to the MSR mixture at 400 °C in the flow MSR experiments might lead to the formation of a stable oxidized phase, which could improve the sintering resistance and long-term stability and potentially stabilize edge sites, even if only the surface-near region is affected. This is suggested by the low total mass increase in the *operando* TGA characterization (Fig. 5).

### 3.3. Redox activation of the Cu–In intermetallic compounds

As we apparently did not achieve *in situ* self-activation of the Cu–In compounds during MSR operation up to 600 °C, we subjected the samples to targeted redox activation, *i.e.*, to alternating cycles of pre-oxidation and pre-reduction treatments. The phase evolution was monitored by *in situ* XRD as well as *in situ* TGA and combined with TEM investigations.

The *in situ* TGA experiments during pre-oxidation in 10 vol% O_2_ are depicted in the ESI† in Fig. S1. We heated CuIn70, CuIn67 and CuIn55 from 25 °C to 800 °C at 5 °C min^−1^ including an isothermal period of 30 min before switching to pure Ar and cooling down, preserving the state reached after the isothermal period. According to the mass increase, the degree of total oxidation of the IMCs to CuO and In_2_O_3_ amounts to approximately one third in all samples (Table S1†). *Ex situ* XRD after the *in situ* TGA experiment (Fig. S2†) confirms the partial oxidation, where we detect varying amounts of Cu^0^ and Cu_2_O, alongside remnants of the intermetallic compounds. The only formed indium phase is In_2_O_3_ and thus, no metallic indium is observed in any sample.

STEM-EDX investigations of the samples before and after the oxidation during the *in situ* TGA treatments reveal a distinct segregation behavior (Fig. S3†). While the untreated catalysts exhibit a homogeneous distribution of Cu and In, formation of In oxide particles and islands (as well as Cu-depleted areas) is observed in the oxidized state.

To gain more insight into the *in situ* formation of a potentially more active IMC–In_2_O_3_ interface, we subjected all samples to alternating oxidation and reduction treatments during *in situ* XRD experiments. This is an important step in redox activation by targeted (partial) decomposition of the intermetallic compound into an active IMC–In_2_O_3_ interface, exhibiting a superior MSR performance compared to Cu/In_2_O_3_.^28^

During the experiments, the samples were periodically shifted in the plane perpendicular to the propagation direction of the beam, increasing the number of crystallites contributing to the diffraction patterns even further. However, this procedure can lead to artifacts caused by the beam hitting the SiC sleeve. Despite these measures, the patterns of untreated CuIn70 before the first transformation appear spotty, meaning that the diffraction rings recorded with the 2D detector are not continuous, but rather consist of individual diffraction spots indicating large crystallites. The reason behind this observation is the texture of the sample, as large crystallites result in spotty diffraction rings, whereas finely dispersed nanocrystals result in continuous Debye rings.

**Fig. 6:**
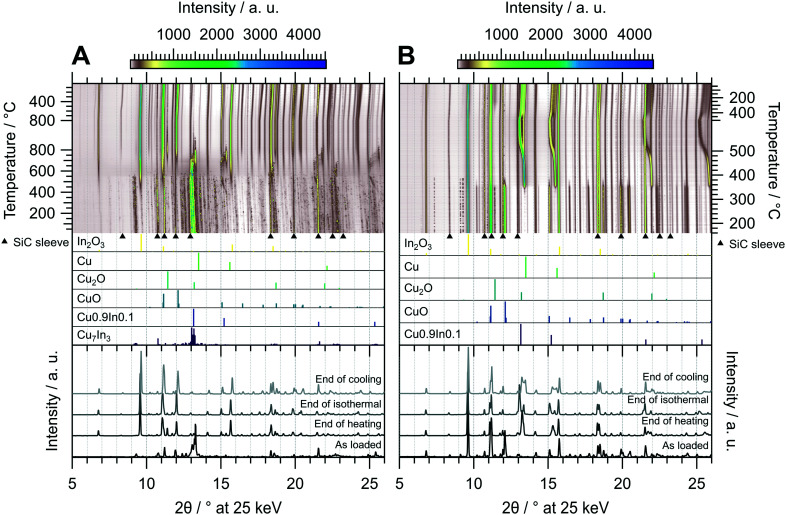
Redox activation of CuIn70 followed by *in situ* XRD. Panel A: Oxidation in 2 ml min^−1^ O_2_ and 8 ml min^−1^ He. Temperature program: heating from 25 °C to 800 °C at a rate of 10 °C min^−1^, followed by an isothermal period of 30 min and cooling to 25 °C at 20 °C min^−1^. Panel B: Reduction in 4 ml min^−1^ H_2_ starting from the state after oxidation. Temperature program: heating from 150 °C to 500 °C at a rate of 10 °C min^−1^, followed by an isothermal period of 30 min and cooling to 25 °C at 20 °C min^−1^. The top panels depict contour plots of the temperature-dependent evolution of the diffraction intensity (color-coded with legend on the top) plotted *vs.* the diffraction angle at the calibrated wavelength (*λ* = 0.4905 Å). In the middle panels, the references for Cu_7_In_3_,^48^ Cu0.9In0.1,^45^ CuO,^49^ Cu_2_O,^50^ Cu (ref. 51) and In_2_O_3_,^52^ calculated with the software *VESTA* 3 (ref. 33) using the respective cif-files, are presented. Additionally, artifacts resulting from the beam hitting the SiC sleeve are marked with black triangles. The bottom panels show selected diffractograms at selected points of the experiment axis.

As demonstrated in Fig. 3, untreated CuIn70 is single-phase Cu_7_In_3_, which is confirmed by the initial diffraction pattern in the *in situ* XRD experiment of the oxidation pre-treatment (Fig. 6 panel A). At around 400 °C, the formation of In_2_O_3_ sets in, accompanied by the segregation of metallic copper. As the reflections of Cu^0^ successively shift to much lower diffraction angles than would be anticipated from simple thermal lattice expansion, progressive incorporation of the larger In into the Cu lattice occurs between 400 °C to 550 °C and causes the formation of a similar composition as Cu0.9In0.1 at 550 °C. At 550 °C, the reflections of Cu_7_In_3_ start to vanish, accompanied by an increase in the background signal in the diffraction angle region of the main reflection, indicating decomposition and melting of the remnants of the IMC. At 700 °C, Cu0.9In0.1 is oxidized to In_2_O_3_ as well, leading to the formation of metallic Cu as well as Cu_2_O and CuO. Successively, formation of Cu_2_O and CuO occurs and we reach full oxidation after 15 min during the isothermal period, where the amount of Cu_2_O saturates. After the oxidative pre-treatment, CuIn70 consists of In_2_O_3_, CuO and traces of Cu_2_O (see bottom panel of Fig. 6 panel A), indicating that oxidation into a Cu–In composite oxide takes place.

Starting the subsequent reduction treatment, the concerted reduction of the Cu_2_O and CuO to metallic Cu starts at 350 °C, yielding Cu^0^/In_2_O_3_ (Fig. 6 panel B). At 480 °C, most of Cu^0^ starts to incorporate indium, leading to the formation of a quasi-continuous array of phases analogous to Cu0.9In0.1. This state essentially represents a substitutional alloy of Cu and indium exhibiting the crystal structure of Cu^0^ with an expanded lattice due to the incorporation of In with a larger atomic radius, with the limiting compositions of elemental Cu and an alloy with close to 10 at% indium. Due to the significantly lower indium content of the alloy compared with Cu_7_In_3_, the decrease of the In_2_O_3_ reflections is hardly detectable. Upon reaching 400 °C in the cooling period, the range of Cu_*x*_In_*y*_ alloys narrows down and the highest indium content is lowered, reducing the separation of the reflections of the limiting compositions. Around the same temperature of 400 °C during the cooling phase, formation of small or highly strained Cu_2_O crystallites sets in, which is indicated by its broad reflections. In summary, the redox activation of CuIn70 led to the formation of Cu0.9In0.1 and In_2_O_3_, providing the aimed for IMC–oxide interface.

**Fig. 7:**
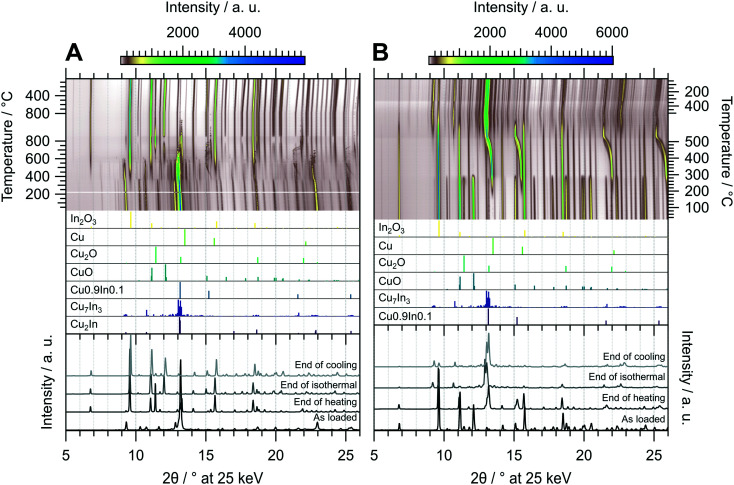
Redox activation of CuIn67 followed by *in situ* XRD. Panel A: Oxidation in in 2 ml min^−1^ O_2_ and 8 ml min^−1^ He. Temperature program: heating from 25 °C to 800 °C at a rate of 10 °C min^−1^, followed by an isothermal period of 30 min and cooling to 25 °C at 20 °C min^−1^. Panel B: Reduction in 4 ml min^−1^ H_2_ starting from the state after oxidation. Temperature program: heating from 25 °C to 500 °C at a rate of 10 °C min^−1^, followed by an isothermal period of 30 min and cooling to 25 °C at 20 °C min^−1^. The top panels depict contour plots of the temperature-dependent evolution of the diffraction intensity (color-coded with legend on the right) plotted *vs.* the diffraction angle at the calibrated wavelength (*λ* = 0.4905 Å). In the middle panels, the references for Cu_2_In,^40^ Cu_7_In_3_,^48^ Cu0.9In0.1,^45^ CuO,^49^ Cu_2_O,^50^ Cu (ref. 51) and In_2_O_3_,^52^ calculated with the software *VESTA* 3 (ref. 33) using the respective cif-files, are presented. The bottom panels show selected diffractograms at selected points of the experiment axis.

The *in situ* XRD experiment of the oxidation treatment of CuIn67 is shown in Fig. 7 panel A. The unidentified phase that decomposed in the MSR test in the recirculating batch reactor is stable in the oxidizing atmosphere up to approximately 350 °C. At this temperature, the initially present Cu_2_In as well as the unidentified phase are both converted to Cu_7_In_3_ in addition to In_2_O_3_. The intermediately formed Cu_7_In_3_ decomposes once more at around 550 °C. A similar phenomenon as in CuIn70 regarding the incorporation of In into the Cu lattice is observed in CuIn67. At around 450 °C, the corresponding reflections appear and continuously shift to lower diffraction angles with increasing temperature, indicating an expansion of the Cu lattice by incorporation of indium, up to approximately 550 °C. Successively, these alloys decompose to Cu^0^ and In_2_O_3_, with the former being oxidized quantitatively during the isothermal period. Cu_2_O as well as CuO start to form concomitant to the decomposition of Cu_7_In_3_ at 550 °C. From approximately 750 °C to 5 min in the isothermal period, Cu_2_O reaches an intermediate maximum concentration, which we explain by comproportionation of CuO and the concurrently converted Cu to Cu_2_O. Then, CuO is re-formed and the amount of Cu_2_O decreases in the isothermal period, again saturating after 20 min during the isothermal period. The resulting phase composition of the sample is very similar to CuIn70 and comprised of In_2_O_3_, CuO and small amounts of Cu_2_O.

Fig. 7 panel B shows the diffractograms of CuIn67 collected during the reductive treatment. Although the qualitative phase composition of CuIn70 and CuIn67 after oxidation is similar, the phase evolution in the subsequent reduction is clearly different. The formation of Cu^0^ starts at 180 °C and continues up to 300 °C, where both Cu_2_O and CuO are quantitatively converted to metallic copper, again yielding a Cu^0^/In_2_O_3_ system. At approximately 380 °C, the incorporation of In into Cu^0^ starts, yielding Cu0.9In0.1 at the start of the isothermal period. The intensity of In_2_O_3_ is progressively reduced from the start of the isothermal period, reaching a minimum at the start of cooling. This increasing availability of indium facilitates the conversion of Cu0.9In0.1 to Cu_7_In_3_ starting after 15 min in the isothermal period. The final state after cooling is composed of the IMC Cu_7_In_3_ with remnants of In_2_O_3_ (see bottom panel Fig. 7 panel B). Hence, the redox activation yielded an interface of Cu_7_In_3_ and In_2_O_3_.

**Fig. 8:**
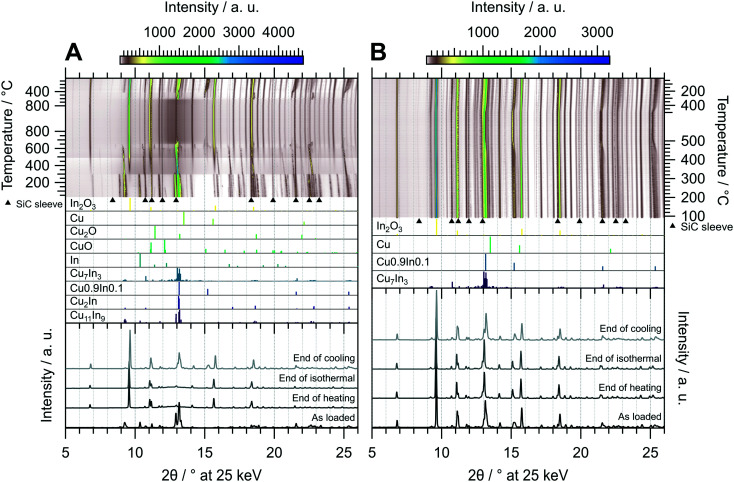
Redox activation of CuIn55 followed by *in situ* XRD. Panel A: Oxidation in 2 ml min^−1^ O_2_ and 8 ml min^−1^ He. Temperature program: heating from 25 °C to 800 °C at a rate of 10 °C min^−1^, followed by an isothermal period of 30 min and cooling to 25 °C at 20 °C min^−1^. Panel B: Reduction in 4 ml min^−1^ H_2_ starting from the state after oxidation. Temperature program: heating from 100 °C to 500 °C at a rate of 10 °C min^−1^, followed by an isothermal period of 30 min and cooling to 25 °C at 20 °C min^−1^. The top panels depict contour plots of the temperature-dependent evolution of the diffraction intensity (color-coded with legend on the right) plotted *vs.* the diffraction angle at the calibrated wavelength (*λ* = 0.4905 Å). In the middle panels, the references for Cu_11_In_9_,^41^ Cu_2_In,^40^ Cu0.9In0.1,^45^ Cu_7_In_3_,^48^ In,^46^ CuO,^49^ Cu_2_O,^50^ Cu (ref. 51) and In_2_O_3_,^52^ calculated with the software *VESTA* 3 (ref. 33) using the respective cif-files, are presented. Additionally, artifacts resulting from the beam hitting the SiC sleeve are marked with black triangles. The bottom panels show selected diffractograms at selected points of the experiment axis.

The oxidative treatment of CuIn55 is highlighted in Fig. 8 panel A. The initial state is composed of the IMC Cu_11_In_9_ and traces of metallic In, which vanishes above 140 °C. No In_2_O_3_ is formed, indicating that In is either incorporated into the IMC or it simply melts (melting point of In = 156.6 °C (ref. 29)). At 300 °C, Cu_11_In_9_ is partially transformed to the high-temperature phase Cu_2_In and the rest melts, observable as a significant increase in the background signal from 2*θ* = 10–15°, while In_2_O_3_ starts to form. At around 490 °C, the background intensity again changes, suggesting that another fraction of the IMC melts. A small amount of Cu0.9In0.1 forms at around 550 °C and vanishes again at 730 °C, while the rest of Cu_2_In melts at 660 °C. The melt remains stable during the isothermal period, resisting oxidation to In_2_O_3_ even at 800 °C. At 620 °C in the cooling period, recrystallization of an unidentified phase next to Cu0.9In0.1 sets in. The former compound vanishes again at 410 °C, giving rise to the formation of Cu_7_In_3_ as well as the formation of a Cu-richer Cu0.9In0.1 phase. The final state after cooling consists of In_2_O_3_, Cu_7_In_3_ and a range of Cu_*x*_In_*y*_ alloys (see bottom panel in Fig. 8 panel A), whereas no Cu_2_O or CuO forms at any stage of the experiment. Thus, the oxidative treatment of CuIn55 already leads to the formation of an IMC–oxide interface, distinguishing it from the respective states of CuIn70 and CuIn67 after the oxidation step of the redox activation.

The reductive treatment of the oxidized CuIn55 is depicted in Fig. 8 panel B and reveals only slight alterations over the course of the entire experiment. At approximately 450 °C, the stoichiometry of the Cu0.9In0.1-analogous phases changes to higher In content. The final state after cooling is composed of In_2_O_3_, Cu_7_In_3_ and various Cu0.9In0.1-analogous alloys, representing an interface of two IMCs with In_2_O_3_, distinguishing CuIn55 from CuIn70 and CuIn67, which feature only one distinct IMC after redox activation (see bottom panel of Fig. 8 panel B).

The ultimate goal of the targeted redox activation was the preparation of intermetallic Cu–In compound/In_2_O_3_ systems. Summarizing the results of the redox activation of the intermetallic Cu–In compounds, all the initially present phases could either be completely decomposed (CuIn70 and CuIn67) or melted and transformed upon recrystallization (CuIn55) during the oxidation treatment. For CuIn70 and CuIn67, which were both quantitatively decomposed to In_2_O_3_, CuO and small amounts of Cu_2_O in the oxidation step, the reductive treatment leads to the re-formation of intermetallic compounds different from their initial ones, however retaining In_2_O_3_ in their final state. Both CuIn70 and CuIn67 exhibit an intermediary Cu/In_2_O_3_ state during the reductive step in accordance with the studies on the reactive metal–support interaction of Cu and In_2_O_3_.^28^ However, in contrast to this study, where Cu_2_In was the main evolving IMC, Cu0.9In0.1 and Cu_7_In_3_ form for CuIn70 and CuIn67, respectively. For CuIn55, we reach the formation of an IMC/In_2_O_3_ interface already after the oxidation step, as two types of intermetallic compounds, namely Cu_7_In_3_ and Cu0.9In0.1, in addition to In_2_O_3_ are present. Hence, the redox activation was successful for all samples, with CuIn70 and CuIn67 consisting of one and CuIn55 of two distinct intermetallic compounds in combination with In_2_O_3_.

We briefly want to focus on the striking feature that apparently Cu_7_In_3_ formed during reduction of CuIn67 is not further converted to Cu0.9In0.1 (as it was the case for the direct reduction of CuIn70). After the oxidation treatment monitored by *in situ* XRD, the qualitative phase composition of CuIn70 (Cu_7_In_3_ before oxidation) and CuIn67 (Cu_2_In before oxidation) is similar, consisting of In_2_O_3_, CuO and traces of Cu_2_O. However, the phase evolution in the oxidation and the quantitative composition are different. It is apparent from the diffractograms that much less In_2_O_3_ remains after the reduction in CuIn67, meaning that more In is available for the formation of the In-richer Cu_7_In_3_ phase. On the other hand, more In_2_O_3_ is retained in the reduction of pre-oxidized CuIn70, leading to the formation of In-poorer Cu0.9In0.1 alloys. The reasons for the different stability of the In_2_O_3_ phase are not obvious, but a different morphology of the samples (form and dispersion of the components) might play an important role next to the difference in total composition. We deem it also unlikely that longer exposure to the reductive atmosphere would lead to the transformation of the newly formed Cu_7_In_3_ to Cu0.9In0.1 alloys in CuIn67. The difference is that in CuIn70, Cu_7_In_3_ is first decomposed to In_2_O_3_, CuO and Cu_2_O in the oxidative treatment and the Cu0.9In0.1 alloys are formed from this composition in the following reductive treatment. In CuIn67, Cu_2_In is decomposed to In_2_O_3_, CuO and Cu_2_O as well, but Cu_7_In_3_ forms in the reduction instead of the Cu0.9In0.1 alloys. It might be possible that the Cu_7_In_3_ phase in CuIn67 could be transformed to Cu0.9In0.1 alloys by repeating the whole cycle (oxidation + reduction), but the evolution of the newly formed Cu_7_In_3_ in CuIn67 upon prolonged exposure to the reduction mixture is unlikely.

### 3.4. MSR performance of the intermetallic compound–oxide interfaces after targeted redox activation

**Fig. 9:**
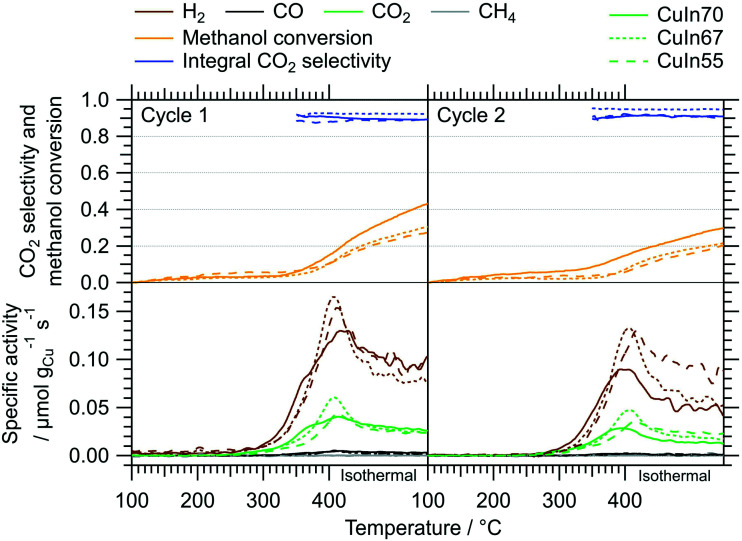
MSR profiles of CuIn70 (solid lines), CuIn67 (dotted lines) and CuIn55 (dashed lines) between 100 °C and 400 °C including an isothermal period of 30 min at 400 °C. Color code: orange – methanol conversion, blue – integral CO_2_ selectivity, specific activity of brown: H_2_, black: CO, green: CO_2_, gray: CH_4_. Heating rate: 5 °C min^−1^; sample mass: CuIn70 – 51.5 mg, CuIn67 – 51.6 mg, CuIn55 – 52.1 mg. Redox-activation in the recirculating batch reactor before the first MSR cycle performed as in the *in situ* XRD experiments.

We elucidated the impact of the redox activation on the MSR performance by applying similar pre-treatment conditions (pre-oxidation in O_2_ up to 800 °C and pre-reduction in H_2_ up to 500 °C, corresponding to the treatments in the *in situ* XRD experiments) in the recirculating batch reactor before the catalytic tests (Fig. 9*vs.*Fig. 2). Table 2 summarizes the catalytic key parameters of the samples without and after one full cycle of redox activation. In contrast to the catalytic tests without pre-treatments (*cf.*Fig. 2), all three catalysts exhibit similar H_2_ and CO_2_ onset temperatures, activity, and CO_2_ selectivity. Differences in the deactivation behavior are evident even in the recirculating batch reactor from the first to the second MSR cycle without any intermediate treatments. CuIn70 displays the strongest deactivation (specific activity toward H_2_ in isothermal period ≈53% in the second cycle) followed by CuIn67 (≈76%), whereas CuIn55 suffers only a minor activity loss. The activity increase, compared to MSR tests without pre-treatments, is also most pronounced for CuIn55 (specific activity for H_2_ increased by a factor of 12). According to *in situ* XRD, CuIn55 is the only sample exhibiting two bimetallic Cu–In compounds (Cu_7_In_3_ and Cu0.9In0.1 alloy structures) in contact with In_2_O_3_ after redox activation. Its structural stability under reducing conditions in the reductive step (Fig. 8 panel B) serves as an explanation for its superior stability in MSR, because a similarly reducing atmosphere is present at high conversions in the batch reactor. CuIn70 consists of a range of Cu_*x*_In_*y*_ alloy structures next to In_2_O_3_ and traces of Cu_2_O after reduction (Fig. 6 panel B). CuIn67 is mainly composed of Cu_7_In_3_ (exhibiting poor long-term stability in the MSR flow test in its untreated state, Fig. 4), with remnants of In_2_O_3_ after pre-reduction (Fig. 7 panel B).

**Table 2:** Comparison of key catalytic parameters of CuIn70, CuIn67 and CuIn55 in MSR with and without redox activation

State	Parameter	CuIn70	CuIn67	CuIn55
Untreated	Onset *T*(H_2_)/°C	260	300	270
	Onset *T*(CO_2_)/°C	280	300	270
	Onset *T*(CO)/°C	380	340	400
	Max. activity H_2_/μmol g_Cu_^−1^ s^−1^	0.018	0.051	0.009
	Max. activity CO_2_/μmol g_Cu_^−1^ s^−1^	0.004	0.015	0.002^a^
	Max. activity CO/μmol g_Cu_^−1^ s^−1^	0.001^a^	0.002^a^	0.001^a^
	*E*_a_ CO_2_/kJ mol^−1^	128.54	122.70	129.56
MSR cycle 1 after redox activation	Onset *T*(H_2_)/°C	230	250	230
	Onset *T*(CO_2_)/°C	230	250	230
	Onset *T*(CO)/°C	280	310	310
	Max. activity H_2_/μmol g_Cu_^−1^ s^−1^	0.130	0.165	0.154
	Max. activity CO_2_/μmol g_Cu_^−1^ s^−1^	0.041	0.061	0.044
	Max. activity CO/μmol g_Cu_^−1^ s^−1^	0.005	0.005	0.005
	*E*_a_ CO_2_/kJ mol^−1^	115.93	118.55	120.57
MSR cycle 2 after redox activation	Onset *T*(H_2_)/°C	190	230	220
	Onset *T*(CO_2_)/°C	190	230	220
	Onset *T*(CO)/°C	270	310	310
	Max. activity H_2_/μmol g_Cu_^−1^ s^−1^	0.090	0.133	0.129
	Max. activity CO_2_/μmol g_Cu_^−1^ s^−1^	0.029	0.047	0.035
	Max. activity CO/μmol g_Cu_^−1^ s^−1^	0.002^a^	0.003	0.002^a^
	*E*_a_ CO_2_/kJ mol^−1^	118.56	119.13	122.83

a Close to noise level, therefore high uncertainty associated.

**Fig. 10:**
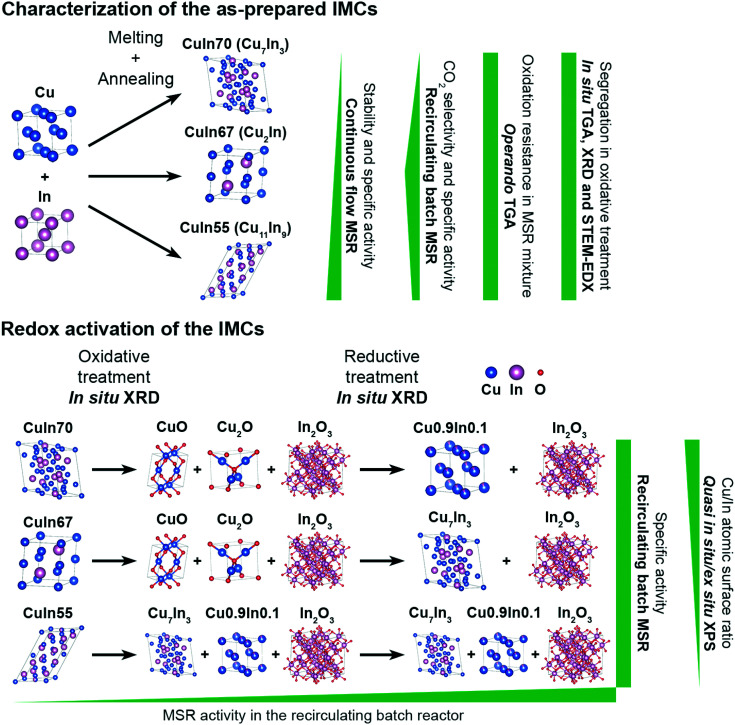
Graphical summary of the most important findings of this study.

To assess the surface chemistry before and after MSR, *ex situ* XP spectra of the state of the redox-activated IMCs after the MSR experiments were recorded and compared with the corresponding spectra of the untreated samples (see ESI† Fig. S4). Note that the spectra of the untreated samples were collected under quasi *in situ* conditions due to the glovebox transfer discussed in the Experimental section. According to the high-resolution Cu 2p and Cu LMM spectra, the IMCs contain mainly intermetallic copper in their untreated state. The In 3d and In MNN spectra indicate the presence of partially oxidized In. After redox activation and two MSR cycles, indium is mostly oxidized in all samples. Copper is mostly present as CuO in CuIn70 after the abovementioned procedure according to both Cu 2p and Cu LMM regions and the atomic Cu/In surface ratio is reduced to 0.25 from initially 1.17 in the untreated state (see Table S2†). In contrast to CuIn70, Cu is still partially metallic in CuIn67 and the surface Cu/In ratio decreases from 1.20 to 0.15. No copper is present in the surface-near region in CuIn55 after redox activation and MSR anymore, implying that an In_2_O_3_ layer covers the sample surface. We emphasize that the exact quantification of oxidized In and Cu phases after the MSR treatment is hampered by the air contact of these samples, but the Cu/In surface ratios are unaffected. Additionally, the XP spectra of the C 1s region of the as-prepared and the post-MSR state depicted in Fig. S5† do not show a clear trend of carbon deposition for the most severely deactivating CuIn70. Therefore, carbon deposition is unlikely the main cause for deactivation. As a graphical overview, all key findings of this study have been illustrated in Fig. 10, visualizing the characterization of the as-prepared samples and the impact of the redox activation on the MSR performance.

## 4. Conclusions

Inspired by the activity increase of a Cu/In_2_O_3_ catalyst at a stable CO_2_ selectivity in MSR upon formation of an Cu_2_In/In_2_O_3_ interface by reactive metal–support interaction, the stability limits, self-activation and methanol steam reforming performance of three distinct intermetallic Cu–In compounds – Cu_7_In_3_, Cu_2_In and Cu_11_In_9_ – were assessed and compared to the performance after a targeted redox activation. The latter allows us to judge the inherent catalytic properties of a Cu–In intermetallic compound–oxide interface resulting from this redox activation.

The stability of the IMCs under MSR conditions appears to be largely independent of the initial stoichiometry, as all three IMCs persist substantial changes during MSR operation. The inherent activity under batch MSR conditions is highest for Cu_2_In, corroborating the results of the Cu_2_In/In_2_O_3_ sample accessed through reactive metal–support interaction. Under flow MSR operation, Cu_7_In_3_ displays considerable deactivation, while Cu_2_In and Cu_11_In_9_ exhibit a stable performance at high CO_2_ selectivity. *Operando* TGA experiments indicated no significant self-activation in terms of partial oxidation for any intermetallic compounds, emphasizing their structural stability.

Redox activation of the Cu–In intermetallic compounds yielded IMC/In_2_O_3_ interfaces with superior MSR performance compared to the untreated samples, although the catalytic profiles appear surprisingly similar. Cu_7_In_3_ converts to Cu0.9In0.1 alloys on In_2_O_3_, Cu_2_In to Cu_7_In_3_ on In_2_O_3_ and Cu_11_In_9_ to Cu_7_In_3_ and Cu0.9In0.1 alloys in contact with In_2_O_3_. Cu_7_In_3_/Cu0.9In0.1/In_2_O_3_ with the highest indium content exhibits the least deactivation, which we explain by formation of stabilizing In_2_O_3_ patches under MSR conditions, alleviating particle sintering and rounding.

Although we demonstrated the proof-of-concept and successful use of targeted redox activation to prepare intermetallic compound–oxide interfaces with superior MSR properties, further studies need to focus on overcoming the inherent drawbacks of unsupported intermetallic compounds. There is a growing need for reliable and reproducible preparation techniques to enhance the surface area, and hence activity, of such intermetallic compounds. A pathway of using dedicated intermetallic compound sputter targets, enabling the preparation of supported intermetallic compound nanoparticles with defined and controlled stoichiometry that are stable under reaction conditions, is one of the most promising synthesis routines so far (*e.g.* as demonstrated by Zimmermann *et al.* employing GaPd_2_ thin films for the selective hydrogenation of acetylene).^53^

Furthermore, the formation of Cu0.9In0.1 analogous alloys in CuIn70 and CuIn55 provides an interesting starting point for future studies. The investigation of the behavior and intrinsic activity of these unordered intermetallic compounds (corresponding to the α phase in the Cu–In phase diagrams in Fig. 1), when present in their unsupported form, would be the logical next step in unraveling the best starting phase for the formation of a highly efficient CuIn–IMC-derived MSR catalyst.

## Conflicts of interest

There are no conflicts to declare.

## Supplementary Material

CY-011-D1CY00913C-s001
