# Supplementary material for: Steering the methanol steam reforming reactivity of intermetallic Cu–In compounds by redox activation: stability vs. formation of an intermetallic compound–oxide interface
Source: Catal Sci Technol. 2021 Jul 23;11(16):5518–33. doi: 10.1039/d1cy00913c (PMC8365629; doi:10.1039/d1cy00913c)
Supplement: CY-011-D1CY00913C-s001 [file CY-011-D1CY00913C-s001.pdf]

Electronic Supplementary Material (ESI) for Catalysis Science & Technology.
This journal is © The Royal Society of Chemistry 2021

Supporting Information

Steering the methanol steam reforming reactivity of intermetallic Cu–In compounds by redox activation: stability *vs.* formation of an intermetallic compound–oxide interface

Kevin Ploner[1], Andrew Doran[2], Martin Kunz[2], Albert Gili[3,4], Aleksander Gurlo[3], Nicolas Köwitsch[5], Marc Armbrüster[5], Johannes Bernardi[6], Maximilian Watschinger[1] and Simon Penner[1,]*

[1]*Department of Physical Chemistry, University of Innsbruck, Innrain 52c, A-6020 Innsbruck*

[2]*Advanced Light Source, Lawrence Berkeley National Laboratory, Berkeley, California 94720, USA*

[3]*Chair of Advanced Ceramic Materials, Institut für Werkstoffwissenschaften und -technologien, Technical University Berlin, Hardenbergstr. 40, D-10623 Berlin, Germany*

[4]*Institute of Chemistry, Technical University Berlin, Sekretariat TC 8, Straße des 17. Juni 124, D-10623 Berlin, Germany*

[5]*Institute of Chemistry, Materials for Innovative Energy Concepts, Technical University Chemnitz, Straße der Nationen 62, D-09111 Chemnitz, Germany*

[6]*University Service Centre for Transmission Electron Microscopy, TU Wien, Wiedner Hauptstr. 8-10, A-1040 Vienna, Austria*

Keywords: copper, indium, decomposition, activity, CO_2 selectivity, *operando* TGA, *in situ* XRD, quasi *in situ* XPS, TEM

*Corresponding Author: simon.penner@uibk.ac.at, Tel: 004351250758003

Section A: Detailed Experimental

1. *In situ* Thermogravimetric analysis (*in situ* TGA)

For the investigation of the oxidation behavior of the ground intermetallic samples, *in situ* TGA was performed in a NETZSCH STA 449F3 Jupiter setup. The system was equilibrated by flushing 30 min with 100 ml min^{-1} Ar (99.999%, Linde) and 30 min with the reaction mixture of 10 ml min^{-1} O_2 in 90 ml min^{-1} Ar, both at 25 °C. The temperature program involved heating to 800 °C at a rate of 5 °C min^{-1}, followed by an isothermal period of 30 min at 800 °C.

2. Transmission electron microscopy (TEM)

Elemental mapping was conducted with EDX spectroscopy in a FEI TECNAI F20 field emission TEM operated at 200 kV. It is equipped with a high angle annular dark-field STEM detector (HAADF), a GATAN GIF Tridiem image filter and an Apollo XLTW SDD X-ray detector, providing a spatial resolution of about 1 nm for EDX maps.

3. Quasi *in situ* X-ray photoelectron spectroscopy (XPS)

Characterization of the surface-near chemical state of the samples was performed with a Thermo Scientific MultiLab 2000 spectrometer equipped with an Alpha 110 hemispherical sector analyzer and a monochromatic Al K_α X-ray gun. The base pressure was kept in the low 10^{-9} mbar range and a flood gun supplying electrons with a kinetic energy of 6 eV was utilized for compensation of sample charging.

Detailed spectra of relevant regions were obtained with a pass energy of 20 eV and an energy step size of 0.05 eV. The background was fitted by a Shirley-type function. The high-resolution spectra of the Cu $2p_{3/2}$ and the In $3d_{5/2}$ regions were utilized for the determination of the surface-near atomic Cu/In ratio. Quantification was based on the relative sensitivity factors[1] and the different inelastic mean free paths derived from the predictive G1 formula by Gries.[2] For the qualitative assignment of chemical species of Cu and In, reference materials were

measured in the same setup under identical conditions. Metallic Cu (sputter-cleaned Cu foil, Goodfellow, ≥ 99.99%), Cu_2O (Sigma-Aldrich, anhydrous, ≥ 99.99% trace metals basis), CuO (Sigma-Aldrich, 99.999% trace metals basis) and $Cu(OH)_2$ (synthesized by precipitation with $CuSO_4 \cdot 5\ H_2O$ (Merck, for analysis, 99.7%) and NaOH (Roth, ≥ 99%)) were chosen for Cu and metallic In (sputter-cleaned In foil, Goodfellow, 99.999%), as well as In_2O_3 (Alfa Aesar, 99.99% metals basis), for In. The untreated intermetallic samples were characterized after crushing in an Ar-filled glovebox and successive transfer under Ar into the XPS chamber, providing quasi *in situ* measurements.[3] The quasi *in situ* conditions could not be realized for the extraction of the samples from the recirculating batch reactor, hence, *ex situ* XPS was employed instead.

Section B: Oxidation pre-treatment monitored by *in situ* TGA as well as post-treatment XRD and TEM

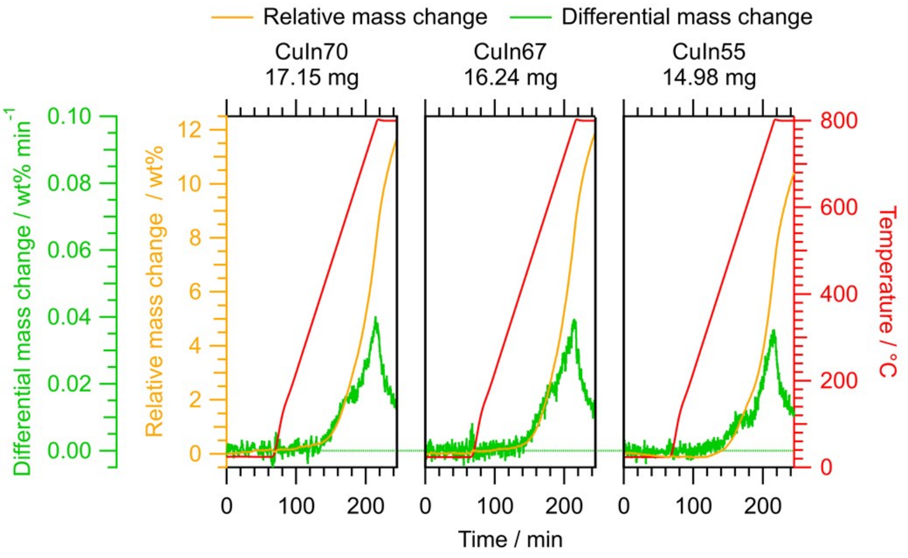

Figure S1: *In situ* TGA investigation of an oxidative pre-treatment of CuIn70, CuIn67 and CuIn55. After flushing for 30 min at 25 °C with 100 ml min^{-1} Ar and 30 min with the oxidative mixture consisting of 10 ml min^{-1} O_2 in 90 ml min^{-1} Ar, the temperature was increased to 800 °C with a rate of 5 °C min^{-1}, followed by an isothermal period for 30 min. The sample masses of each sample are stated above the respective panels.

Table S1: Evaluation of the mass increase determined by *in situ* TGA.

	CuIn70	CuIn67	CuIn55
Mass increase / wt%	11.7	11.9	10.4
Fraction of total oxidation to CuO and In_2O_3 / %	36.0	36.1	29.7

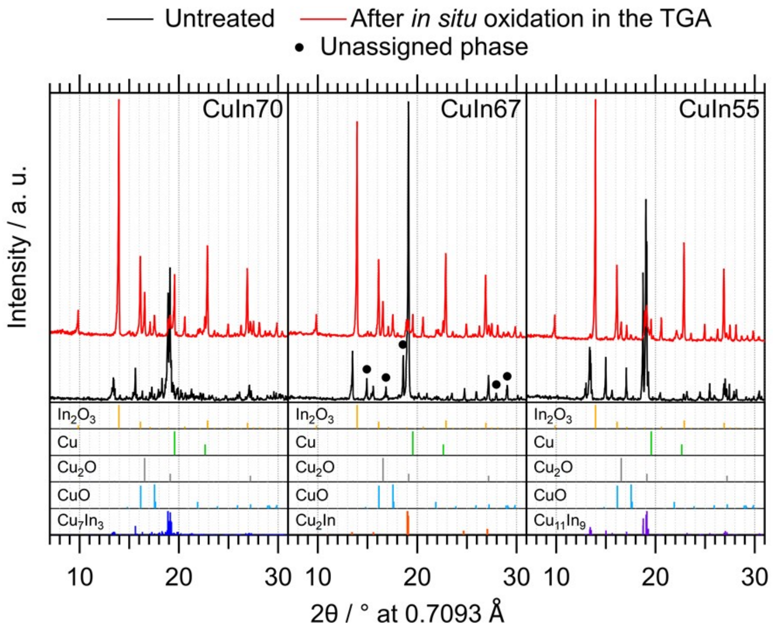

Figure S2: *Ex situ* XRD patterns of CuIn70, CuIn67 and CuIn55 after the oxidative *in situ* TGA treatment depicted in Figure S1. The references for Cu,[4] Cu_2O,[5] CuO[6] and In_2O_3[7] were taken from the ICDD database[8] and the references for Cu_7In_3,[9] Cu_2In[10] and $Cu_{11}In_9$[11] were calculated with the software *VESTA* 3[12] using the respective cif-files.

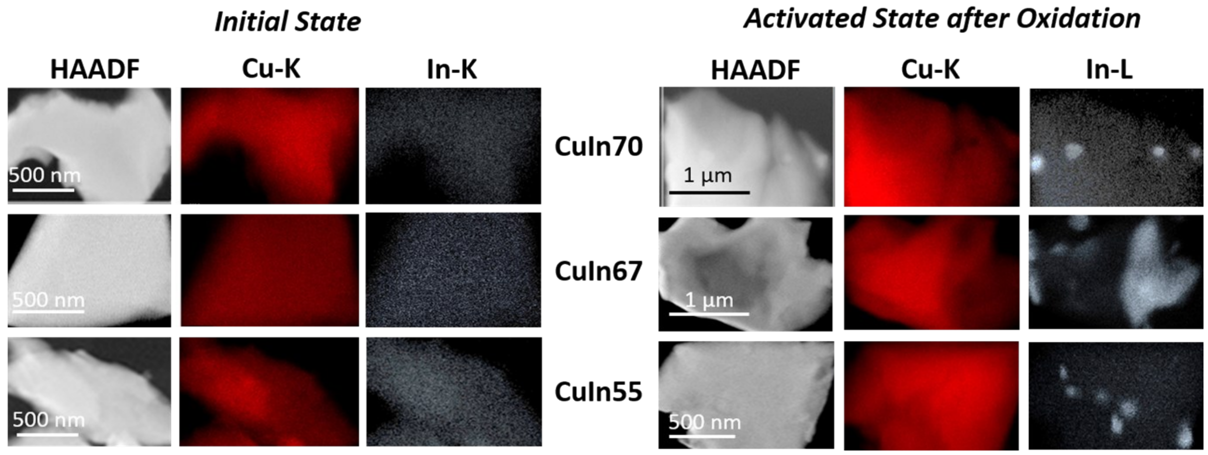

Figure S3: STEM-EDX characterization of the initial states (left Panel) and states after oxidation (right Panel) CuIn70, CuIn67 and CuIn55. HAADF images alongside the respective EDX maps of the Cu- K (red) and the In-K/In-L (blue) intensity are shown for each sample. The oxidation was carried out by *in situ* TGA under the conditions as described in the context of Figure S1.

Section C: *Quasi in situ* and *ex situ* XPS of the IMCs

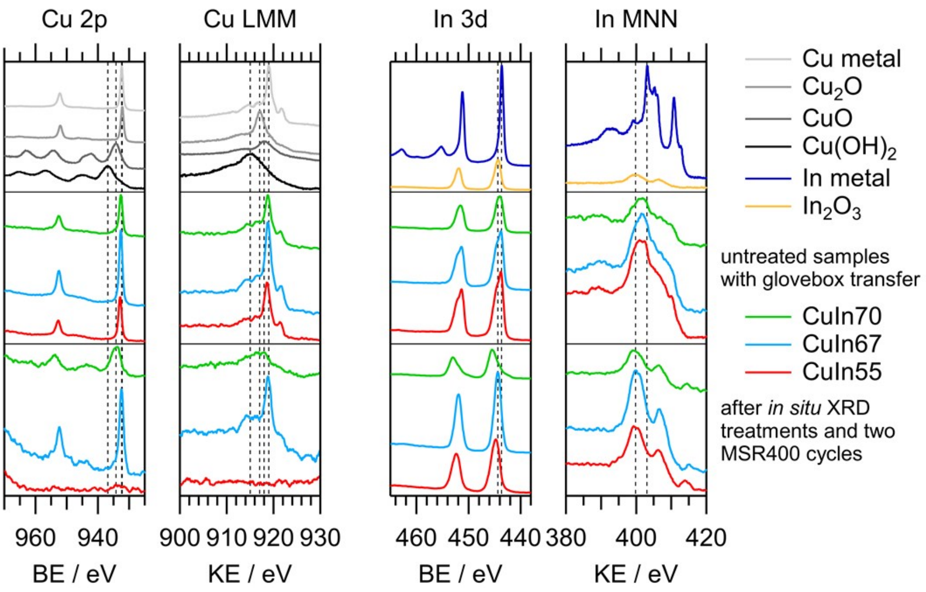

Figure S4: High-resolution Cu 2p and In 3d XP spectra (plotted *vs.* BE = binding energy) and Cu LMM and In MNN Auger regions (plotted *vs.* KE = kinetic energy) of untreated CuIn70, CuIn67 and CuIn55 and after the redox activation observed by *in situ* XRD followed by two MSR cycles up to 400 °C (*cf.* Figure 9 in the main text).

Table S2: Cu/In surface ratio according to XPS.

	CuIn70	CuIn67	CuIn55
Cu/In ratio untreated	1.17	1.20	0.64
Cu/In ratio after oxidation, reduction and two MSR cycles	0.25	0.15	0.01*

*Cu 2p signal in the range of the noise, hence, ratio is not significant and set to zero.

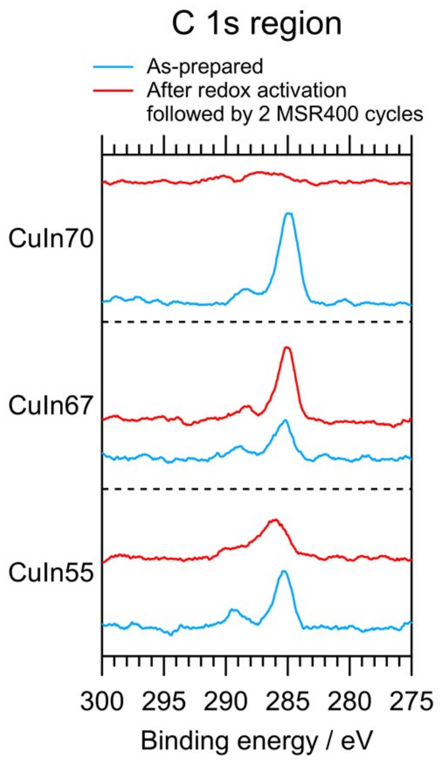

Figure S5: XP spectra of the C 1s region of CuIn70, CuIn67 and CuIn55 as-prepared (blue traces) and after redox activation followed by two MSR400 cycles (red traces). The spectra of one catalyst are depicted on the same scale to guarantee the relative comparability of the as-prepared and the post-MSR states. The spectra have been smoothed to improve the visibility of characteristic features.

References

1 *CasaXPS. Processing Software for XPS, AES, SIMS and More*, Casa Software Ltd.

2 W. H. Gries, A Universal Predictive Equation for the Inelastic Mean Free Pathlengths of X-ray Photoelectrons and Auger Electrons, *Surf. Interface Anal.*, 1996, **24**, 38–50. DOI: 10.1002/(SICI)1096-9918(199601)24:1<38::AID-SIA84>3.0.CO;2-H.

3 M. Watschinger, K. Ploner, D. Winkler, J. Kunze-Liebhäuser, B. Klötzer and S. Penner, *Operando* Fourier-transform infrared–mass spectrometry reactor cell setup for heterogeneous catalysis with glovebox transfer process to surface-chemical characterization, *Rev. Sci. Instrum.*, 2021, **92**, 24105. DOI: 10.1063/5.0041437.

4 H. E. Swanson and E. Tatge, *Natl. Bur. Stand.(US) Circ. 539*, 1953, **1**, 15.

5 A. Kirfel and K. Eichhorn, Accurate structure analysis with synchrotron radiation. The electron density in Al_2O_3 and Cu_2O, *Acta Crystallogr., Sect. A: Found. Crystallogr.*, 1990, **46**, 271–284. DOI: 10.1107/S0108767389012596.

6 S. Asbrink and A. Waskowska, CuO: X-ray single-crystal structure determination at 196 K and room temperature, *J. Phys.: Condens. Matter*, 1991, **3**, 8173–8180. DOI: 10.1088/0953-8984/3/42/012.

7 H. E. Swanson, N. T. Gilfrich and G. M. Ugrinic, *Natl. Bur. Stand.(US) Circ. 539*, 1955, **5**, 26.

8 *ICDD Database PDF-4+*, International Centre for Diffraction Data, Newtown Square, PA 19073, USA, 2010.

9 A. S. Koster, L. R. Wolff and G. J. Visser, Structure of Copper–Indium Cu_7In_3, *Acta Crystallogr., Sect. B: Struct. Sci.*, 1980, **36**, 3094–3096. DOI: 10.1107/S0567740880010886.

10 G. C. Che and M. Ellner, Powder Crystal Data for the High-Temperature Phases Cu_4In, Cu_9In_4(h) and Cu_2In(h), *Powder Diffr.*, 1992, **7**, 107–108. DOI: 10.1017/S0885715600018340.

11 T. P. Rajasekharan and K. Schubert, Kristallstruktur von $Cu_{11}In_9$, *Z. Metallkd.*, 1981, **72**, 275–278.

12 K. Momma and F. Izumi, *VESTA 3* for three-dimensional visualization of crystal, volumetric and morphology data, *J. Appl. Crystallogr.*, 2011, **44**, 1272–1276. DOI: 10.1107/S0021889811038970.
